# Identification of an Amino Acid Metabolism Signature Participating in Immunosuppression in Ovarian Cancer

**DOI:** 10.1155/2022/4525540

**Published:** 2022-06-22

**Authors:** Hanlin Yang, Dan Zi

**Affiliations:** ^1^Department of Gynaecology and Obstetrics, The Affiliated Hospital of Guizhou Medical University, Guizhou Medical University, Guiyang 550004, China; ^2^Department of Gynaecology and Obstetrics, Guizhou Provincial People's Hospital, Guiyang 550002, China

## Abstract

Ovarian cancer is one of the most fatal gynecologic cancer types, and its heterogeneity in the microenvironment limited the efficacy of the current treatment. In this study, we aimed at building a risk score to predict patient survival based on the amino acid metabolic genes and TCGA RNA-seq dataset (*n* = 376). We first used univariate analysis and PCA to select and test the survival-related genes, and the LASSO regression was applied to build the risk score signature with prediction accuracy estimation by survival analysis and ROC. We then conducted GSEA and GSVA to investigate the biological roles of the signature and run ESTIMATE and 4 different immunocyte infiltration algorithms to investigate the immunological diversity between the risk groups. Furthermore, the immune checkpoint expression was compared. We finally explored the cMap and PRISM database to screen out sensitive drugs for high-risk patients and analyzed the oncogenic role of *TPH1* by clone formation and transwell migration assays. As a result, the risk score predicted patients' survival and stage with high accuracy. We found that the signature mainly affected the extracellular activities and cancer immunity by functional enrichment. We further discovered that the high-risk OV harbored a high level of stromal cell infiltration and was associated with highly infiltrated fibroblasts and decreased CD8+ T cells. The immune checkpoint analyses showed that *TGFB1* and *CD276* were upregulated. Finally, we screened out 4 PRISM drugs with lower IC_50_ in the high-risk group and validated the oncogenic role of *TPH1* in OV cancers. We believe this research offered a novel understanding of the interplay between amino acid metabolism and immunity in OV and will benefit patients with better prognostic management and therapeutic strategy development.

## 1. Introduction

Ovarian (OV) cancer is one of the most lethal cancer types for female health, and it is the second fatal solid tumor of gynecologic cancers [1]; the routine treatment of OV is the combination of cytoreductive surgery and chemotherapy based on the platinum usage. Approximately 10% of OV cancers are familial syndromes, and 90% of them are sporadic [2]. The major risk factors for OV cancer are family history and the carrier of *BRCA1/BRCA2* mutations; mean lifespan risk for OV cancer is 30% in *BRCA1* mutation carriers and 27% in *BRCA2* mutation carriers [2]. In recent years, the morbidity and mortality rates of OV cancer were slowly decreasing, but it still threats the health of females, giving rise to the exploration of more effective cancer characteristic prediction and therapeutic strategies.

Metabolism reprogramming is a common feature in various cancer types that adapts cancer cells to the energy of substrates requirement of rapid proliferation or metastasis. Glycolysis has been noticed as the leading metabolic form of OV cancer [3], and many strategies were developed to target the glycolysis network to treat OV cancer. However, the effects vary due to the heterogeneity of the cancer histology and microenvironment. Amino acid metabolism is also a critical metabolic activity in cancers; it not only provides the substrate for protein production but also intersects the purine and one-carbon metabolism to fulfill the various biomass requirement of cancer cells [4]; the many amino acids have been discovered to promote cancer progression and metastasis [5].

Amino acid metabolism can not only affect the proliferation of cancer cells themselves but also regulate the noncancer cells in the tumor microenvironment. Arginine is a critical substrate that functions during macrophages' M1 and M2 polarization; M1 macrophages activate iNOS, a cancer-supporting factor [6, 7], to catalyze arginine, producing NO to attack cancer cells, whereas M2 macrophages express arginase 1, converting arginine to ornithine to further promote the cancer cells [5]. Tryptophan deprivation can inhibit the stabilization of T cells [8], and its catabolic product, kynurenine, can prevent the maturation of T17 cells and stimulate the regulatory T-cell proliferation [9]. However, the association between amino acid metabolism and cancer immunity remained unclear in OV cancer.

In this study, we aimed at constructing a risk score based on the amino acid metabolism-related gene sets to predict the OV cancer patient survival and clinical stage. We will also investigate the association between amino acid metabolism and the immune landscape in the tumor microenvironment using functional analyses and various algorithms. We believe that this study will provide a new perspective on the OV pathological mechanism, and benefit patients with better prognostic management and novel therapeutic target development.

## 2. Materials and Methods

### 2.1. Sequencing Data Collection

The sequencing data and the corresponding clinical information on ovarian cancers were obtained from The Cancer Genome Atlas (TCGA). The downloaded FPKM expression matrix was then transformed into the TPM matrix. The amino acid metabolism gene list was obtained from the GOBP_CELLULAR_AMINO_ACID_METABOLIC_PROCESS of the Gene Ontology (GO). The samples without clinical survival information were removed. The gene sets for functional analyses were retrieved from the Molecular Signatures Database (MSigDB) of the Gene Set Enrichment Analysis (GSEA).

### 2.2. Principal Component Analysis and Risk Score Construction

The 286 amino acid metabolism-related genes were first tested by univariate cox regression to select the survival-associated candidates, and the genes that passed the test were used for principal component analysis (PCA) clustering to divide the samples into 2 clusters. After PCA clustering, we compared the prognostic diversity of the clusters by conducting a survival analysis. Furthermore, the least absolute shrinkage and selection operator (LASSO) regression [10] was applied to select the parameters from the genes that passed the univariate test and build a risk score for predicting the survival risks of the OV patients. The “lambda.min” was selected for obtaining the model with the lowest deviance, and the corresponding coefficients were also presented. The risk score was organized as follows:

(1) $$\begin{array}{c} \text{Risk score}=\sum_{i}^{n}\beta_{i}*g_{i}. \end{array}$$

The augment *g*_*i*_ refers to the expression of the gene *i* selected by LASSO and *β*_*i*_ means the coefficient of gene *i*.

Besides, the expression differences of the survival-related amino acid metabolism genes between different risk groups were presented in a heatmap. And the expression differences of the amino-acid-metabolism-related pathways were analyzed by gene set enrichment analysis (GSEA) and gene set variation analysis (GSVA) [11].

We estimated the prognostic value of the risk score by conducting a survival analysis for the patients separated by the median risk score, and receiver operating characteristics curves (ROC) were utilized to access the accuracy of the survival prediction of 1-year, 3-year, and 5-year by the risk score, and the prediction accuracy was compared among risk score, PCA cluster, and age.

### 2.3. Clinical Significance of the Risk Score and the Association between the Classifiers

Apart from the ability for predicting survival, we also evaluate the association between stages, age, and risk score. The risk level was compared between three stages and the age groups (separated by the median age of 59). Also, the ROC was applied to estimate the prediction accuracy of clinical stages by the risk score, PCA cluster, and age.

Subsequently, we performed unsupervised clustering of all the ovarian cancer samples by consensus clustering using the function “ConsensusClusterPlus” of the R package “ConsensusClusterPlus.” We selected the best *k* value by evaluating the cumulative distribution function (CDF) and the relative change in area under CDF curve. Survival analysis was used to access the prognostic significance of the consensus clusters, and a Sanky plot was drawn to explicit the distribution of the data flow among risk groups, PCA cluster, and the consensus clusters.

### 2.4. Functional Analysis of the Transcriptional Diversity between Risk Groups

To clarify the biological changes caused by the amino acid metabolism signature, we conducted functional enrichment analyses using the gene sets from GO and Kyoto Encyclopedia of Genes and Genomes (KEGG) datasets. Gene set enrichment analysis (GSEA) was conducted to calculate the enrichment score of the biological processes from GO or the pathways from KGG. Meanwhile, gene set variation analysis (GSVA) [11] was performed to compare the gene sets' variation between the high-risk and low-risk groups.

The stemness diversity of all samples was calculated from the signature (mRNAsi, EREGnRBAsi) calculated by innovative one-class logistic regression (OCLR) algorithm [12], and it was compared between the risk groups.

### 2.5. Immune Landscape and Immunocyte Infiltration Diversity between Risk Groups

Since the functional enrichment analyses had indicated the involvement of immune-related biological processes and pathways, we first investigate the immune landscape of the two risk groups by Estimation of STromal and Immune cells in MAlignant Tumor tissues using Expression data (ESTIMATE) analysis using the R package “estimate” [13], and the ESTIMATEScore, ImmuneScore, StromalScore, and TumorPurity were compared respectively between the high-risk and low-risk group. As for the immunocyte infiltration diversity, we utilized 4 different approaches to firmly evaluate the infiltrating levels of various immunocytes between the two groups by the function “deconvolute” of the R package “immunedeconv” [14]. A heatmap (scaled by rows) and box plots were presented to visualize the differences.

### 2.6. Stimulating and Inhibitory Immune Checkpoint Expression

The immune analyses suggested the immunosuppressive roles of high risk. We further explored whether immune checkpoints were employed to facilitate the depression of antitumor immunity. The expression of the 20 inhibitory and 35 stimulatory immune checkpoints [15] between the high-risk and the low-risk groups was compared and visualized by a heatmap and box plots. To further validate the correlation between immune checkpoints and amino acid metabolism, the enrichment levels of the amino-acid-metabolism-related gene sets in the samples ranged by immune checkpoint expression levels were analyzed by GSVA.

### 2.7. Antitumor Drug Development for Patients with High Risk

To develop a novel therapeutic strategy for high-risk patients, we searched the connective map (cMap) and PRISM compound databases to screen out high-sensitivity drugs. The top 50 compounds were presented in a heatmap showing the opposite similarity between the effects of the compounds and the transcriptional changes caused by the amino acid metabolism signature on 9 cancer cell lines. The mechanism of action (MoA) of these compounds, which showed the effective mechanisms of drugs, were presented in a scatter plot. The 50% inhibitory concentration (IC_50_) of drugs in the PRISM database for all ovarian cancer patients was predicted by the “callPhenotype” function of the R package “oncoPredict” [16], using the input of a training cell line expression matrix and a response matrix and the ridge regression algorithm. The top 4 significant drugs with IC_50_ lower than 30 were analyzed, and their IC_50_ was compared between the high-risk and the low-risk groups.

### 2.8. Cell Culture, Small-Interfere RNA Knockdown, and Western Blot Detection of the Protein Levels of OV Cells

The A2780 cells were cultured in 10% FBS containing the RPMI-1640 medium under 37°C and 5% CO_2_. The knockdown of *TPH1* was performed using small-interfere RNAs with transfection reagents. After transfection for 48 hours, the cells were harvested and lyzed using RIPA lysis buffer. After ultrasound sonication and centrifugation, the proteins were heated with loading buffer in a mental bath. The electrophoresis and membrane transferring were conducted according to the standard protocol. The membranes were blocked with 5% skim milk powder and incubated with the primary antibodies overnight under 4°C. After secondary antibodies incubation, the membranes were washed, and the bands were detected using chemiluminescence. Each experiment was performed three times.

### 2.9. Clone Formation, Transwell Cell Migration, and Invasion Assay

After the transfection of 48 h, the cells were harvested and cultured at a low concentration (2000 cells per well) in a 6-well plate. After 14 days, the cells were washed with PBS and stained using crystal violet. We counted the effective clones to describe the clone formation ability of the cells. For transwell assay, the cells were resuspended in RPMI-1640 medium containing 1% FCS and added to the upper chamber with a concentration of 1 × 10^4^ cells per chamber. The lower room was filled with RPMI-1640 medium with 20% FBS. For invasion assay, each chamber was coated with Matrigel diluted in the medium before cells were planted. After incubation for 24 hours, the cells were stained with crystal violet for 1 hour, and the cells in the upper chamber were erased using a cotton swab. Each experiment was performed three times.

### 2.10. Statistical Analyses

All the statistical analyses were run on the R software. The mutual correlations of the genes were quantified by Pearson's correlation coefficient. All the survival analyses were examined by log-rank test, and the ROC was used to calculate the AUC of the predictions. The risk, gene expression, and immunocyte infiltration level differences between different groups were tested by Student's *t*-test or Wilcox test. Grouped comparison was tested by ANOVA (for normally distributed variables) or the Kruskal–Wallis test (for non-normally distributed variables), and Dunnett's multiple comparisons test were applied as post hoc test for ANOVA. *P* < 0.05 was considered statistically significant. ^*∗*^, ^*∗∗*^, and ^*∗∗∗*^ referred to the *P* value less than 0.05, 0.01, and 0.001 respectively.

## 3. Results

### 3.1. PCA Clustering and Construction of an Amino Acid Metabolism-Related Risk Score

**Figure 1:**
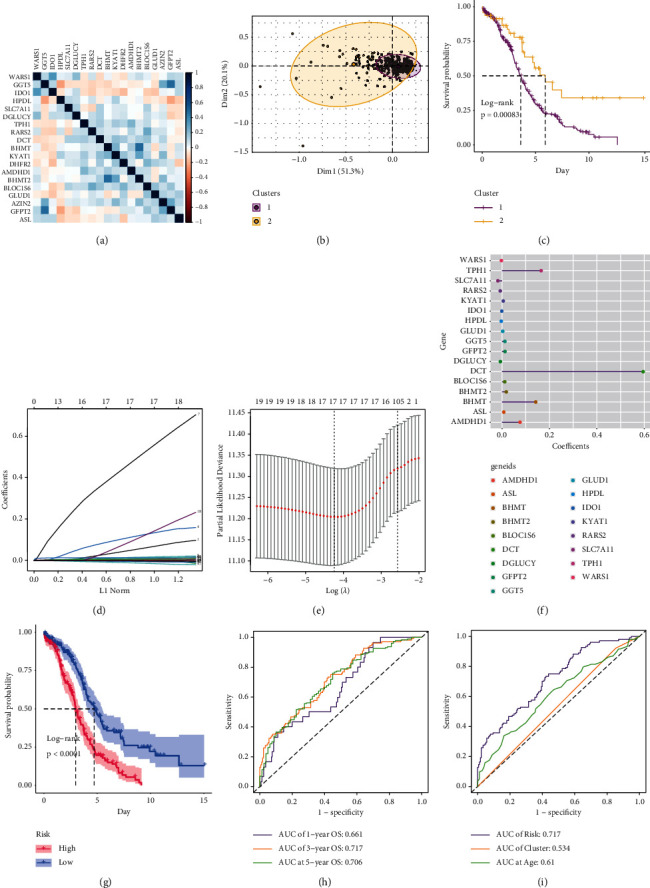
PCA clustering and risk score construction using LASSO (a) The mutual correlations of the genes that passed the univariate cox analysis. (b) The PCA clustering of the patients based on the genes in Figure 1(a). (c) The Kaplan–Meier curves show the ability of the PCA clusters 1 and 2 in separating patient overall survival rate. (d) LASSO coefficients profiles. (e) LASSO deviance profile for selecting the best numbers of parameters. (f): Coefficients of the retained predictors. (g) Kaplan–Meier curve shows the survival prediction ability of the risk score. (h) ROC presents the predictive accuracy of 1-, 3- , 5-year overall survival of the risk score. (i) Comparison of the survival predictive accuracy between risk score, PCA cluster, and age. PCA, principal component analysis; LASSO, the least absolute shrinkage and selection operator; ROC, receiver operating characteristic curve.

In order to identify a survival-related amino acid metabolism signature for OV patients, we downloaded a gene list of 286 amino acid metabolism from MsigDB of GSEA, and univariate Cox regression was applied to screen out survival-related genes. As a result, 19 genes were significantly associated with patient survival and their mutual correlations were presented in Figure 1(a); most of the genes shared low correlations. The genes that passed univariate analysis were then used to conduct PCA clustering, 2 clusters were identified, and the patients grouped in cluster 1 suffered a lower survival rate (Figures 1(b) and 1(c)).

Subsequently, the LASSO regression was used to select parameters to construct a risk score, and 17 genes were retained with the lambda.min value of 0.0144 (Figures 1(d) and 1(e)). The coefficients of the 17 genes are presented in Figure 1(f), 14 genes were identified as risky genes, and 3 were protective genes. According to the median risk score, all patients were classified into high-risk or low-risk groups. We validated that the amino-acid-metabolism-related genes that passed the univariate analysis were differentially expressed between the risk groups, as *IDO, WARS1, RARS2, HPDL, DGLUCY,* and *SLC7A11* were highly expressed in the low-risk group, and the remaining were highly expressed in the high-risk group, which was consistent with the results of their coefficients (Supplementary Figure S1A). The GSEA and GSVA of the amino-acid-metabolism-related gene sets exhibited a series of elevated pathways (Supplementary Figures S1B, S1C).

To test the risk score's ability to predict the survival rate, survival analysis was conducted on the patients in the high-risk and low-risk groups, and the high-risk group exhibited a significantly lower survival rate (Figure 1(g)). The prediction accuracy was examined by ROC, and the risk score can predict patient 1-year, 3-year, and 5-year survival rate with the area under curve (AUC) values of 0.661, 0.717, and 0.706, respectively, and the predictive performance of the risk score outperformed PCA cluster and age (Figures 1(h) and 1(i)), indicating that the risk score harbored a high prognostic value.

### 3.2. High Risk Associated with Higher Cancer Stage, High Age, and PCA Cluster 1

**Figure 2:**
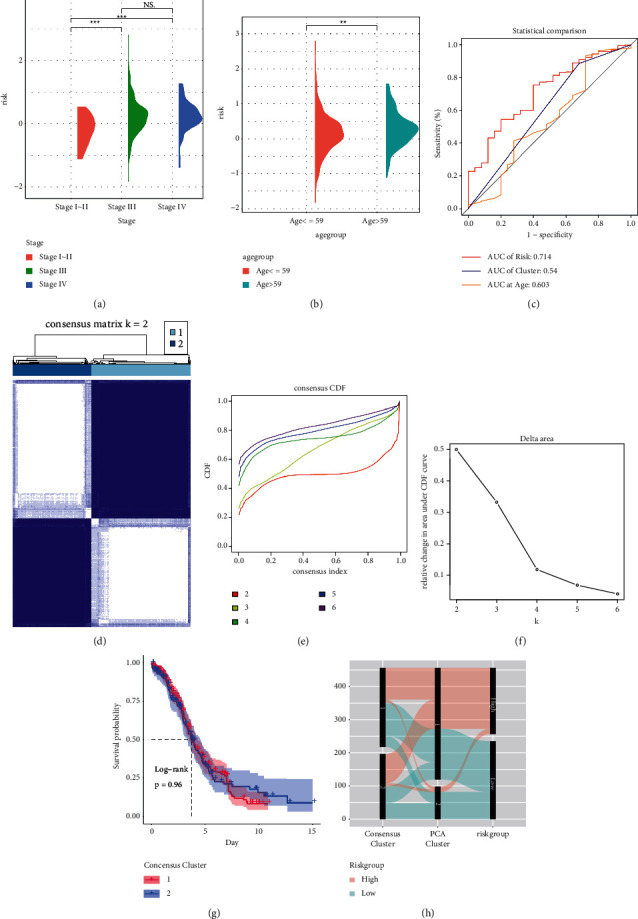
Clinical relevance of the risk score. (a) and (b) The semi-violin plots exhibit the risk differences between stages (a) and age groups (b). (c) ROC estimates the predictive accuracy of the stage by risk score, PCA cluster, age. (d)–(f) The consensus clustering results were presented by the consensus matrix (d), consensus CDF of different *k* value (e), relative change in area under CDF (f). (g) Kaplan–Meier curve testing the survival predictive ability of the consensus clusters. (h) The sanky plot exhibited the data distribution between PCA cluster, consensus cluster, and risk group. ROC, receiver operating characteristic curve; PCA, principal component analysis; CDF, cumulative distribution function.

The clinical significance of the risk score, except for survival rate, was then explored. As depicted in Figures 2(a) and 2(b), samples with stages III and IV, and age above 59 years exhibited higher risk compared to stages I∼II and the low age group. The accuracy of stage prediction by risk score, PCA cluster, and age was evaluated by ROC, and the risk score showed the highest AUC value (Figure 2(c)).

Additionally, we conducted unsupervised classifying using consensus clustering, and all the samples were divided into 2 clusters according to the best *k* value (Figures 2(d)–2(f)). However, the consensus clusters did not separate the survival rate of patients (Figure 2(g)). The correlations between risk groups, PCA clusters, and consensus clusters were visualized in a Sanky plot, and almost all the high-risk distributions flowed to PCA cluster 1 (Figure 2(h)).

### 3.3. Amino Acid Metabolism Was Involved in Extracellular Biological Activities in OV

**Figure 3:**
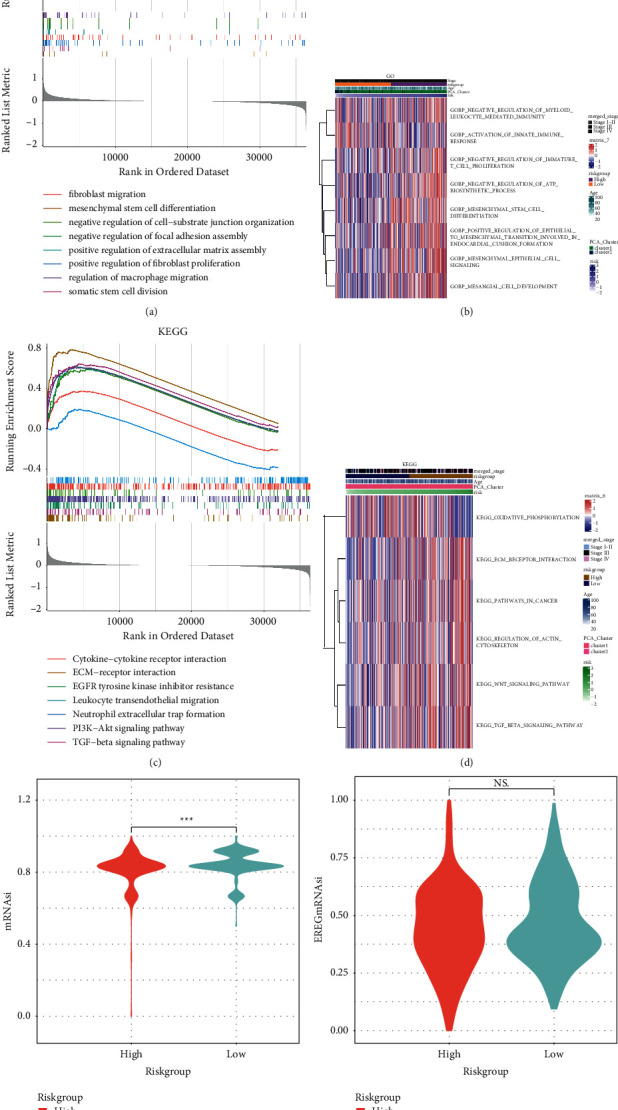
Functional analyses and stemness index calculation. (a) and (b) GO biological processes functional enrichment by GSEA (a) and GSVA (b). (c) and (d) KEGG pathways functional enrichment by GSEA (c) and GSVA (d). (e) and (f) Comparison of stemness between risk groups by the calculation of mRNAsi (e) and EREGmRNAsi (f). GO, gene ontology; KEGG, kyoto encyclopedia of genes and genomes; GSEA, gene set enrichment analysis; GSVA, gene set variation analysis.

To investigate the biological roles of amino acid metabolism in OV, we conducted GSEA and GSVA of the biological processes from the GO database and the pathways from the KEGG database. For the GO biological processes, we noticed that mesenchymal stem cell, fibroblast, and macrophage-related activities were highly enriched in the high-risk group, and the cell adhesion was negatively regulated (Figure 3(a)). Similarly, the GSVA results of GO showed mesenchymal cell-related pathways. Notably, the GSVA results also presented down-regulated immunity in the high-risk group (Figure 3(b)). As for the enrichment results from KEGG, we found the activated TGF-*β* signaling pathway and ECM-receptor interaction via both GSEA and GSVA. Moreover, the GSVA of KEGG discovered the active regulation of the cytoskeleton. These results suggested that the high amino acid metabolism status of OV may facilitate the interaction between the mesenchymal stem cell differentiation, fibroblasts, and cancer cells, and led to immunological changes and cell migration.

We then calculated the stemness of the OV samples and compared it between the risk groups. We noticed that the mRNAsi was lower in the high-risk group, indicating the stem cell differentiation in the high-risk group, although EREGmRNAsi was not significantly different (Figures 3(e) and 3(f)).

### 3.4. The High Risk of OV Was Related to High Cancer-Associated Fibroblasts and Low CD8+ T-Cell Infiltration

**Figure 4:**
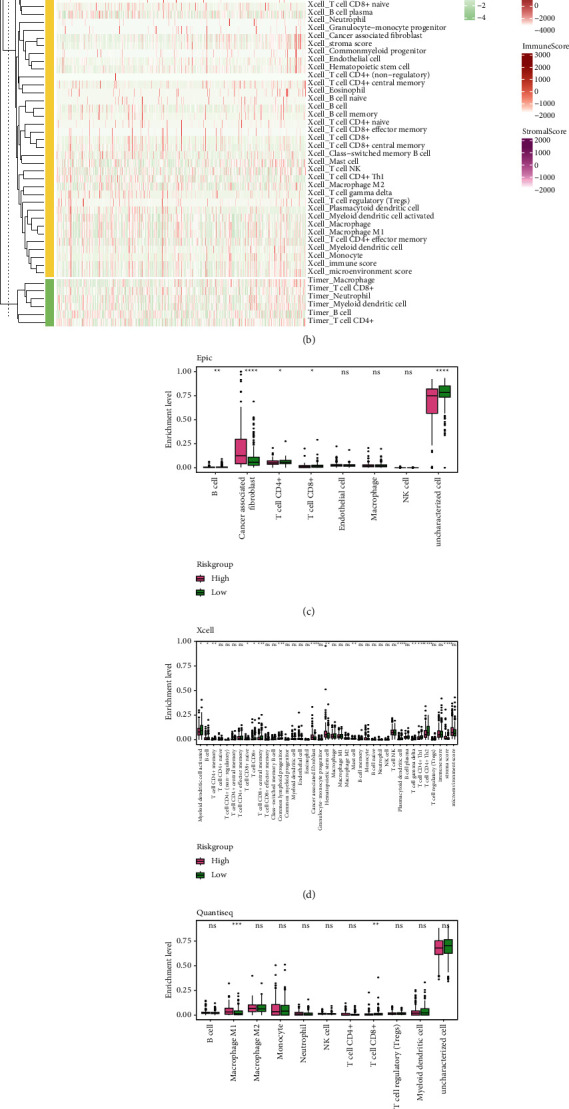
ESTIMATE and immunocyte infiltration. (a) comparison of ESTIMATEScore, ImmuneScore, StromalScore, and TumorPurity between the risk groups. (b) A heatmap shows the immunocyte infiltration by QuanTIseq, Epic, Xcell, and timer algorithms. (c)–(f) Box plots displayed the comparison of different immunocyte enrichment levels between risk groups by epic (c), Xcell (d), QuanTIseq (e), timer (f). ESTIMATE, Estimation of STromal and immune cells in MAlignant Tumor tissues using Expression data.

To seek the diversity of the immune status caused by the amino acid metabolism signature, we first calculated the ESTIMATE of the samples. As presented in Figure 4(a), the high-risk group showed higher ESTIMATEScore, StromalScore, and lower TumorPurity. We employed 4 different approaches to calculate the immunocyte infiltration to go further with the immune infiltration. The heatmap of the immunocyte results exhibited that cancer-associated fibroblast infiltration, identified by both the Xcell and Epic algorithms, increased as the risk grew (Figure 4(b)). We then conducted statistical comparisons of immunocyte levels between the high-risk and low-risk groups. The cancer-associated fibroblasts were significantly enriched in the high-risk group according to the Epic and Xcell approaches (Figures 4(c) and 4(d)). Besides, we noticed that the enrichment of CD8+ T cells was lower (Figures 4(c)–4(e)) and macrophages were higher in the high-risk group (Figures 4(d)–4(f)). The immune landscape of the OV indicated that the immunity was affected.

### 3.5. High Risk Was Associated with a Higher Level of CD276 and TGFB1

**Figure 5:**
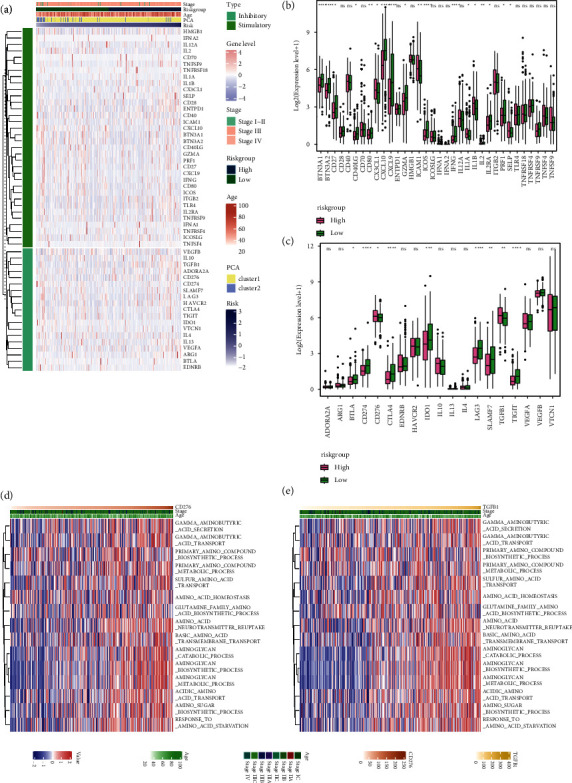
Immune checkpoints expression level comparison. (a) A heatmap shows the 55 immune checkpoints expression level of each sample. (b) and (c) Comparison of the 35 stimulatory (b) and 20 inhibitory (c) immune checkpoints expression differences between risk groups. (d) and (e) The expression pattern between CD276 (d), TGFB1 (e), and the amino-acid-metabolism-related pathways presented by heatmaps.

Immune checkpoints have been discovered as critical factors contributing to immunity depression. Hence, we compared the expression of the immune checkpoint between the risk groups. As the heatmap showed, many stimulatory immune checkpoints seemed to decrease as the risk grew, including *CXCL10, BTN3A1, BTN3A2, CD40LG, GZMA, PRF1, CD27, CXCL9, IFNG, CD80,* and *ICOS,* while the inhibitory immune checkpoints *TGFB1* and *CD276* seemed to increase (Figure 5(a)). The expression of those molecules was then compared between the risk groups. As exhibited in Figure 5(b), the expression of *BTN3A1, BTN3A2, CD27, CD40LG, CD80, ICOS, IFNG, IL2, IL2RA,* and *PRF1* was decreased in the high-risk group. As for inhibitory immune checkpoints, we noticed that *CD276* and *TGFB1* were upregulated in the high-risk group, suggesting their roles in mediating immune suppression. To further confirm the association between amino acid metabolism and *CD276* and *TGFB1*, we analyzed the amino-acid-metabolism-related gene set enrichment levels in samples ranged by *CD276* and *TGFB1*, respectively. As presented in Figure 5(d) and Figure 5(e), the enrichment levels of these gene sets increased as the expression of *CD276* and *TGFB1* was elevated.

### 3.6. Development of Novel Drugs Targeting the High-Risk OV

**Figure 6:**
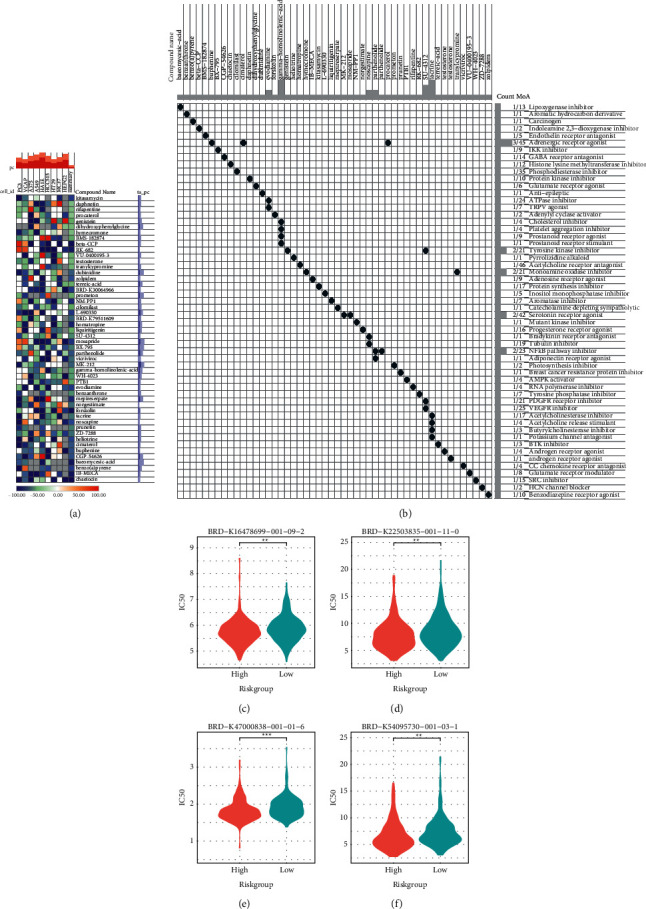
Development of risk-sensitive drugs to OV patients. (a) and (b) A heatmap presents the top 50 cMap compounds causing opposite transcriptional disturbance to that caused by the median risk (a) and the scatter plot shows their corresponding MoA (b). (c) Comparison of the IC_50_ levels between the risk groups of 4 drugs from PRISM database. cMap, connective map; MoA, mechanism of action; PRISM, profiling relative inhibition simultaneously in mixtures.

For the high-risk OV patients, we sought for more sensitive compounds to treat them. The cMap online tool was employed to analyze the transcriptional changes that arose from the median risk score, the top 50 compounds with opposite transcriptional disturbance to those that arose by median risk score were presented in a heatmap, and their MoA was also shown (Figures 6(a) and 6(b)). The most associated compound was chaetocin, a histone lysine methyltransferase inhibitor, and three of the compounds were adrenergic receptor agonists.

We further explored the PRISM database for potential sensitive drugs, the top 4 drugs with the most remarkable fold change and IC50 less than 30 were selected, and their IC50 was compared between the high-and low-risk group, where the drug BRD-K47000838-001-01-6 showed the lowest IC50 in the high-risk group (Figures 6(c)–6(f)).

### 3.7. Knockdown of TPH1 Expression Depressed the Clone Formation, Migration, and Invasion Ability of OV Cancer Cells

**Figure 7:**
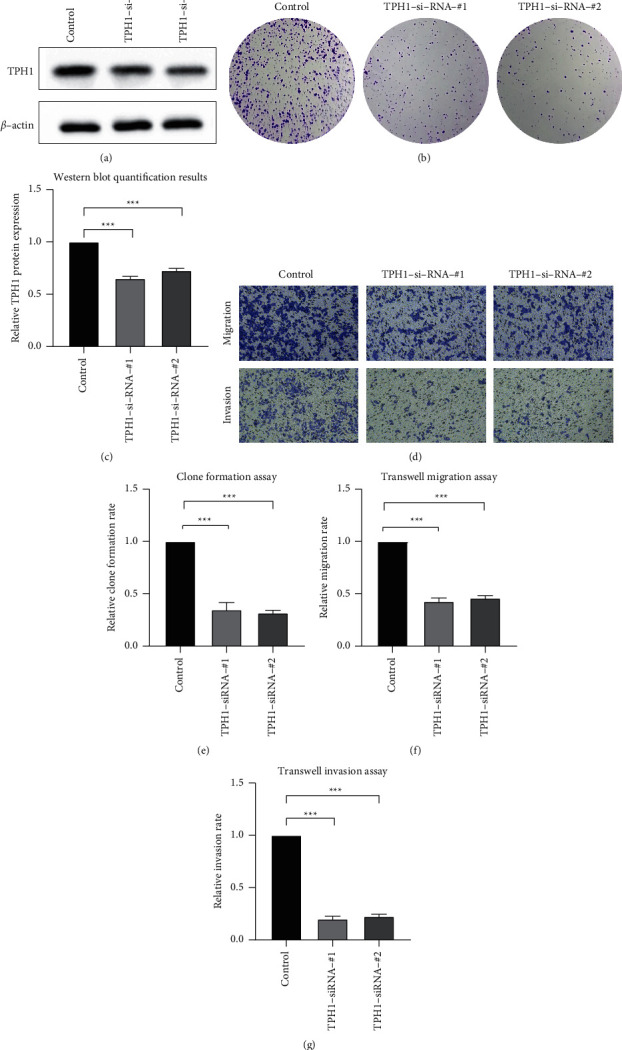
The effects of TPH1 knockdown of the clone formation, migration, and invasion ability of OV cancer cells (a): Western blot results in the control, TPH1-si-RNA-#1, TPH1-si-RNA-#2 groups. (b) The quantification results of protein expressions. (c) and (d) Images of the clone formation assay (c), transwell migration (upper panel), and invasion (lower panel) assay (c) results in the control, TPH1-si-RNA-#1, and TPH1-si-RNA-#2, respectively. (e)–(g) The quantification results of the clone formation assay (e), transwell migration assay (f), and the transwell invasion assay (g) in the control, TPH1-si-RNA-#1, and TPH1-si-RNA-#2 groups, respectively.

We performed the western blot detection of the protein levels in control, TPH1-si-RNA-#1, and TPH1-si-RNA-#2 groups. As exhibited in Figures 7(a) and 7(b), the protein levels in TPH1-si-RNA-#1 and TPH1-si-RNA-#2 groups were decreased. The clone formation assay results showed that the clone assay of OV cancer cells in the TPH1-si-RNA-#1 and TPH1-si-RNA-#2 groups was depressed (Figure 7(c)). Similarly, the transwell results also presented that TPH1 knockdown inhibited the OV cancer cell migration and invasion (Figure 7(d)). The quantification results of these experiments are shown in Figures 7(d)–7(g). These results implied the oncogenetic roles of TPH1 in OV cancers.

## 4. Discussion

Amino acid metabolism controls the protein synthesis of cells, which are the most critical components in cellular activities. Here, we built a risk score for OV patients based on the amino acid metabolism signature using LASSO.

Previously, many metabolism-related cancer risk scores have been reported to predict the overall survival of OV patients, including total metabolism, energy metabolism, and lipid metabolism signature. However, the roles of amino acid metabolism in affecting OV patients' survival have not been explored so far. Herein, we first established an amino acid metabolism-based risk score in OV cancers and revealed its roles in patient survival. This risk score can predict patient survival, especially long-term survival, with high accuracy, outperforming the previous metabolism-related models [17, 18]. And similarly, it can predict the clinical stage of OV cancers with high performance as well. The diversity of the expression pattern of the amino-acid-metabolism-related genes and gene sets validated the association between the risk score and amino acid metabolism.

Amino acid metabolism plays an important role in regulating anticancer immunity in many cancers. As the previous studies found, the metabolites of tryptophan metabolism supported tumor-associated macrophages to facilitate immunosuppression in pancreatic cancer [19]. High levels of arginase that catalyze the L-arginine can inhibit the proliferation of antigen-specific T cells in lung cancer [20]. Besides, serine and glycine metabolism can destroy the anticancer function of macrophages and neutrophils [21]. In OV cancers, only glutamine was found as a key molecule in modulating myeloid-derived suppressor cells (MDSCs) activities, and targeting glutamine can ease the immunosuppressive effects led by MDSCs [22], and the evidence of the association between amino acid metabolism and cancer immunosuppression is still lacking. In this study, we discovered the potential association between highly infiltrated cancer-associated fibroblasts, decreased CD8+ T cells, and the amino acid signature, and to our knowledge, this is the first study revealing this association.

For the potential underlying mechanism, fibroblasts were found to utilize the extracellular lactate, which promoted their amino acid biosynthesis and inhibited the tricarboxylic acid (TCA) cycle [23]. The acidification of the microenvironment was attributed mainly to the glycolysis of cancer [24], which transferred the cancer cells from TCA to macromolecule metabolism and lactate production. This may also be one of the factors explaining the association between amino acid metabolism and fibroblasts in the tumor microenvironment. But importantly, amino acid biosynthesis, such as glycine and proline, directly provided the substrates for extracellular collagen production and assembly, and this may result from the glycolysis in fibroblasts themselves [25]. Apart from collagen production, fibroblasts were also found to provide necessary fuels (such as lactate, amino acids, and fatty acids) to cancer cells, and this process was facilitated by cancer cell-derived paracrine oxidative stress [26]. Shortly, the amino acid metabolism triggered by the glycolysis inside the fibroblasts or the lactate stimulation from cancer cell glycolysis promoted the extracellular matrix production and fuel provision for cancer cells, leading to immunity exclusion and cancer growth support. A study has reported that the inhibition of a metabolism enzyme of the tryptophan of fibroblast restored the T-cell response in vivo [27], and this was in accordance with our finding that CD8+ T cells were decreased in the high-risk group, indicating that T cells were the main immunosuppressive target of fibroblasts driven by abnormal amino acid metabolism.

For the molecular discoveries of the high-risk disturbance in OV cancer, we noticed the elevated expression of TGFB1. This discovery further confirmed the involvement of fibroblasts in high-risk OV cancer, since TGFB1 is the driver of collagen accumulation produced by fibroblasts and immune suppression, and the inhibition of TGF-*β* signaling pathway and expression of *LOXL2* depressed the fibroblast activities and pathological collagen accumulation [28]. Moreover, the inhibition of TGF-*β*1 improved the function of CD8+ T cells [29]. These suggested that abnormal amino acids employed TGF-*β* signaling pathway to control the microenvironment alteration. Interestingly, we also noticed the elevated *CD276* expression in the high-risk group. *CD276* has been reported to increase *HIF-1α* expression and promote the glycolysis of cancer cells [30]. This provided a hypothesis that the high-risk OV cancers highly expressed *CD276* to enhance glycolysis, which directly stimulated the amino acid metabolism of fibroblast or acidated the microenvironment to prompt fibroblast metabolism. At the same time, they expressed *TGFB1* to promote fibroblast activities, and as a result, both the two pathways may support tumor growth and depress normal immunocytes, like T cells. For the association between the two immune checkpoints and amino acid metabolism, only a recent study has demonstrated that glutamine metabolism inhibition decreased *CD276* expression and enhanced granzyme B produced from CD8+ T cell via ROS [7]. And there is no evidence of the correlation between amino acid metabolism and *TGFB1*. Hence, our study presents a novel mechanism of immune checkpoints-mediated effects on OV cancers.

In this study, we finally confirmed the oncogenetic role of *TPH1* in OV cancer. *TPH1* catalyzed the reaction of enhanced degradation of tryptophan to serotonin, and its oncogenetic has been discovered in breast cancer, bladder cell carcinoma, and colon cancer [31–33]. Similarly, we found that the knockdown of TPH1 inhibited OV cancer clone formation and migration ability, which is consistent with previous findings. Interestingly, TPH1 participated in mast cell-mediated immunosuppression, indicating its potential role in tumor-supporting and immunity depression [33]. However, this requires further investigation.

## 5. Conclusion

Comprehensively, we identified a novel risk score based on OV cancer amino acid metabolism, the risk score predicted survival and tumor stages with high accuracy, and the oncogenic role of the risky gene *TPH1* was experimentally validated. We also discovered the immunological roles of amino acid metabolism signature. The high-risk group was associated with increased cancer-associated fibroblast infiltration and decreased CD8+ T cells. Besides, the immune checkpoints *CD276* and *TGFB1* were highly expressed. We believe this study sheds light on the understanding of the association between amino acid metabolism and immunosuppression in OV cancer. It will benefit patients with better prognostic management and provide novel targets for developing more effective therapeutic strategies.

## Data Availability

The TCGA expression data and clinical information can be accessed at https://portal.gdc.cancer.gov/. The gene list of amino acid metabolism can be downloaded from https://www.gsea-msigdb.org/gsea/msigdb/cards/GOBP_CELLULAR_AMINO_ACID_METABOLIC_PROCESS.html. The GO and KEGG gene sets for functional analyses can be retrieved from https://www.gsea-msigdb.org/gsea/msigdb/index.jsp.

## Supplementary Materials

Supplementary Materials Supplementary Figure S1. The association between the risk groups and the amino-acid-metabolism-related gene set expression. A: The heatmap shows the expression diversity of the amino-acid-metabolism-related gene that passed the univariate analysis between the risk groups. B, C: enrichment and variation differences of the amino-acid-metabolism-related gene sets between the risk groups by GSEA (B) and GSVA (C).
