# Supplementary figures and images for: Identification of an Amino Acid Metabolism Signature Participating in Immunosuppression in Ovarian Cancer
Source: Evid Based Complement Alternat Med. 2022 Jun 22;2022:4525540. doi: 10.1155/2022/4525540 (PMC9242802; doi:10.1155/2022/4525540)

A

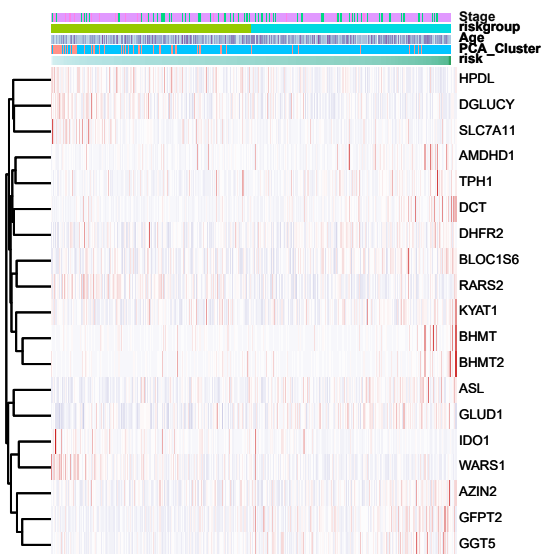

B

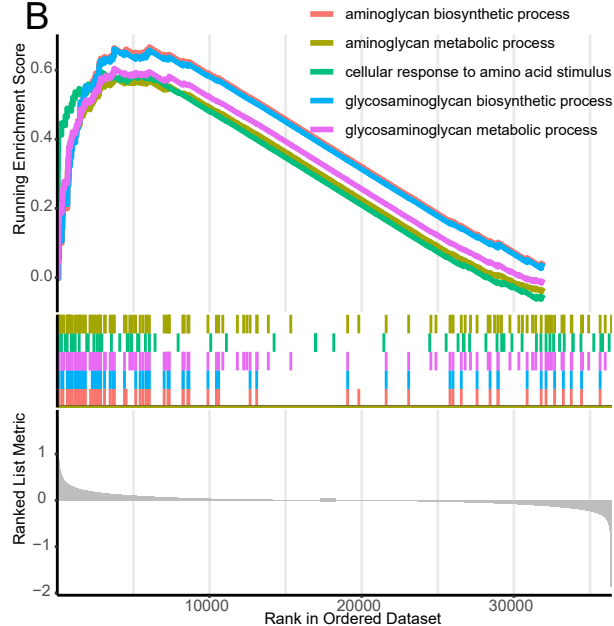

C

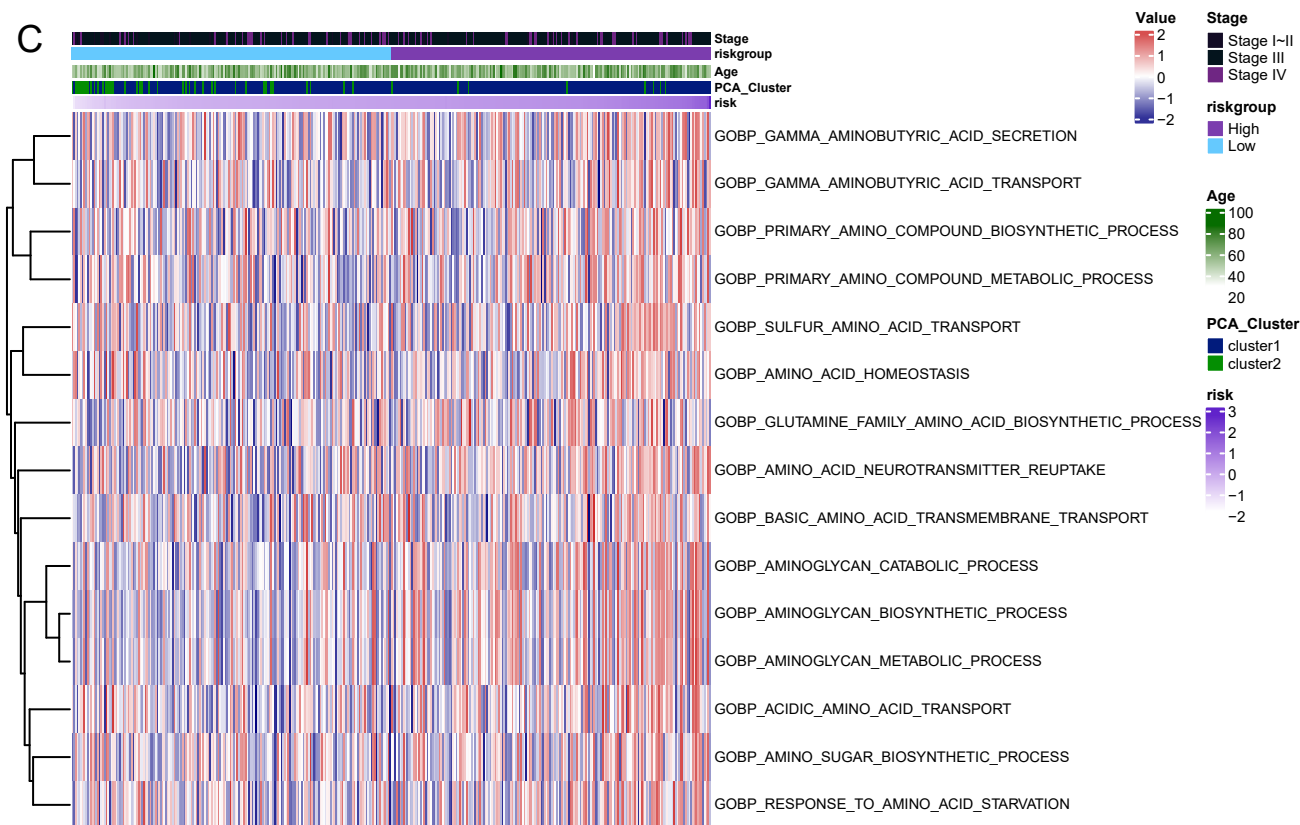

Supplement: Supplementary Materials — Supplementary Figure S1. The association between the risk groups and the amino-acid-metabolism-related gene set expression. A: The heatmap shows the expression diversity of the amino-acid-metabolism-related gene that passed the univariate analysis between the risk groups. B, C: enrichment and variation differences of the amino-acid-metabolism-related gene sets between the risk groups by GSEA (B) and GSVA (C). [file 4525540.f1.pdf]
